# Dietary Puerarin Modulates the Rumen Microbiota and Enhances Growth Performance and Rumen Fermentation in Hu Sheep

**DOI:** 10.3390/ani16152425

**Published:** 2026-08-05

**Authors:** Chuntao Nie, Jiaxin Ye, Daibo Fu, Xinqi Pan, Tangkai Zhu, Wenkai Chen, Miaomiao Bai, Xiaozhen Song

**Affiliations:** 1 Jiangxi Province Key Laboratory of Animal Nutrition and Engineering Research Center of Feed Development, Jiangxi Agricultural University, Nanchang 330045, China; 16668228134@163.com (C.N.); yejiaxin0206@163.com (J.Y.); m13330126185@163.com (X.P.); 18970475191@163.com (T.Z.); 15018477084@163.com (W.C.); 2 Jiujiang Academy of Agricultural Sciences, Jiujiang 332000, China; fudaibo0612@163.com; 3 College of Animal Science, Ganzhou Polytechnic, Ganzhou 341008, China

**Keywords:** puerarin, Hu sheep, 16S rRNA sequencing, rumen microbiota, nutrient digestibility, rumen fermentation

## Abstract

Puerarin has been suggested to improve ruminant productivity, but its effects on rumen fermentation and rumen microbiota in sheep remain insufficiently understood. In this study, Hu sheep were fed diets supplemented with 0, 50, 100, 200, 400, or 800 mg/kg puerarin for 30 d to evaluate growth performance, apparent nutrient digestibility, serum biochemical and antioxidant indices, rumen fermentation, and rumen microbial community structure. Puerarin supplementation improved growth performance, with the 100 mg/kg group showing the greatest average daily gain. Acid detergent fiber digestibility was also significantly increased in the 100 mg/kg group. In the rumen, microbial crude protein concentration was significantly increased in the 100 and 400 mg/kg groups, propionate concentration was significantly increased in the 50 and 100 mg/kg groups, and the acetate-to-propionate ratio was significantly decreased in the 100 mg/kg group. In serum, albumin and glucose concentrations were significantly increased at appropriate supplementation levels, whereas malondialdehyde was reduced in the 100 mg/kg group but increased in the 800 mg/kg group. 16S rRNA sequencing showed limited effects on overall bacterial diversity, but changes in specific rumen microbial taxa and predicted microbial functions were observed. Collectively, these findings suggest that dietary puerarin, particularly at 100 mg/kg, may improve growth performance in Hu sheep, at least in part, through improving rumen fermentation.

## Simple Summary

Plant-based feed additives are being explored as safe ways to improve animal production, but their effects in sheep are still not fully understood. In this study, we tested puerarin, a natural compound from kudzu root, in the diets of Hu sheep to see whether it could improve growth, feed use, rumen function, and health-related blood indicators. Sheep were fed diets containing different amounts of puerarin for 30 days. The results showed that puerarin improved daily weight gain, and the best overall response was observed at 100 milligrams per kilogram of diet. At this level, sheep also showed better digestion of fiber, more favorable rumen fermentation, higher levels of useful fermentation products and microbial protein, and better blood indicators related to metabolism and oxidative balance. Analysis of rumen bacteria showed that puerarin did not strongly change overall bacterial diversity, but it was associated with changes in some bacterial groups and their predicted functions. Overall, these findings suggest that puerarin may be a promising natural feed additive for improving nutrient use, rumen efficiency, and growth performance in Hu sheep.

## 1. Introduction

Driven by global population growth and evolving dietary preferences, mutton and lamb have emerged as indispensable protein sources in the human diet. There is growing consumer demand for high-quality, safe, and efficiently produced mutton. Hu sheep are a prominent indigenous Chinese breed that plays a pivotal role in the national mutton production industry. This breed is characterized by exceptional reproductive performance and efficient growth characteristics, and is highly valued by consumers for its superior carcass traits and nutritional value [1]. In recent years, the substantial demand for meat products has driven the rapid development of China’s indigenous meat sheep breeding industry. In the current market-driven industry, the lamb sector profits significantly from raising lambs that yield high-quality meat at lower costs [2]. Therefore, numerous studies have focused on enhancing growth and productivity in sheep [3,4]. Currently, confined feeding systems are widely adopted in most large-scale meat sheep farms. Under this management model, sheep primarily derive nutrients from basal diets. However, beyond a certain age, the growth rate of sheep tends to decline, reducing economic returns for producers [5]. In ruminants, feed efficiency and growth performance are closely linked to rumen function, because the rumen is the primary site of microbial fermentation, feed degradation, the production of volatile fatty acids (VFAs), and the synthesis of microbial crude protein (MCP) [6]. Thus, nutritional interventions capable of modulating rumen microbial composition and improving rumen fermentation may represent an effective approach to enhancing productivity and health in sheep.

Phytoestrogens have attracted considerable attention because of their potential to improve animal performance and physiological status [7,8]. Due to structural and functional similarities with the estrogenic hormone 17β-estradiol, isoflavones are recognized as natural phytoestrogens [9]. Soy and red clover isoflavones are among the most extensively studied in animal production. Previous studies have shown that these compounds can improve growth performance and modify rumen microbial composition [10,11]. Compared with these commonly studied isoflavones, puerarin is a distinctive C-glycoside isoflavone extracted from the root of kudzu (*Pueraria lobata*) [12]. The C-glycosidic bond confers metabolic stability against β-glucosidase hydrolysis, providing significant advantages over O-glycosylated analogs [13]. In contrast to the predominantly O-glycosidic forms found in soy and red clover isoflavones, puerarin differs in absorption pattern, metabolic fate, and bioavailability, which may result in distinct biological effects. Compared to the direct inclusion of *Pueraria lobata* root material or crude extracts, the use of a standardized puerarin product with defined purity allows more accurate control of the supplementation dose and reduces potential confounding effects from other phytochemical constituents. A comparative study in rats showed that *Pueraria lobata* crude extract and isolated puerarin differed in their antioxidant properties and modulatory effects on hepatic cytochrome P450 enzymes, indicating that constituents other than puerarin in crude preparations may exert additional biological effects [14]. Therefore, the use of standardized puerarin enables a more specific evaluation of its biological effects in ruminants.

Accumulating evidence has shown that puerarin supplementation can improve feed efficiency and productive performance, enhance antioxidant capacity, and support intestinal and immune function, thereby contributing to improved livestock productivity [15,16,17]. However, current research has predominantly focused on monogastric animals (e.g., pigs, poultry) or murine models; data regarding the application of puerarin in ruminants remain markedly limited. In particular, the effects of puerarin on rumen fermentation and rumen microbial ecology in sheep are still poorly understood. Because rumen microorganisms are directly involved in feed degradation, fermentation end-product formation, and microbial protein synthesis, modulation of the rumen microenvironment may represent an important mechanism by which puerarin enhances ruminant performance [18]. Our previous studies showed that dietary puerarin supplementation alleviated heat stress-induced impairment of beef quality in cattle and was associated with improved antioxidant capacity and remodeling of the rumen microbial community [19]. These findings suggest that puerarin may serve as a promising nutritional regulator of rumen function in ruminants. Nevertheless, the dose-dependent responses of growing lambs to puerarin have not been systematically evaluated, leaving the optimal inclusion level undefined.

Therefore, the present study was conducted to systematically investigate the effects of dietary puerarin supplementation across a wide dose range (0 to 800 mg/kg) on growth performance, nutrient digestibility, serum biochemical and antioxidant indices, rumen fermentation characteristics, and rumen bacterial composition in Hu sheep. We hypothesized that puerarin may improve growth performance and nutrient utilization by optimizing rumen fermentation and affecting rumen microbial composition, thereby exerting beneficial effects on the productivity and physiological status of Hu sheep.

## 2. Materials and Methods

### 2.1. Animals, Experimental Design, and Diets

Puerarin (purity ≥ 90%) was procured from Shaanxi Herbpure Co., Ltd. (Xi’an, China). A single-factor completely randomized design was adopted. Seventy-two 4-month-old male Hu sheep (21 ± 0.9 kg) were randomly allocated to a control group and five puerarin supplementation groups, with dietary inclusion levels of 50, 100, 200, 400, and 800 mg/kg on a dry matter (DM) basis, respectively. Each group contained four replicates, with three sheep per replicate. A 20-day adaptation period was provided to allow the lambs to acclimate to the experimental environment and gradually transition to the experimental diets, thereby minimizing stress and digestive disturbances before formal data collection. Following a 20-day adaptation period, a 30-day feeding trial was conducted to evaluate the dose-dependent short-term responses of growing Hu sheep to dietary puerarin supplementation. The control group received a basal diet, while the treatment groups were provided with the same basal diet supplemented with five graded levels of puerarin in the concentrate portion. Sheep were fed twice daily (08:00 and 18:00) with ad libitum access to water. The amount of feed provided was adjusted daily based on their intake, ensuring a 10% surplus. The dietary concentrate-to-forage ratio was 40:60 (DM basis), and peanut vine was chopped to an average length of approximately 8–10 cm before mixing. Because puerarin was included at relatively low dietary levels, it was first thoroughly incorporated into the concentrate portion to facilitate uniform premixing and reduce the risk of uneven distribution. The puerarin-containing concentrate was subsequently blended in batches with chopped peanut vine to prepare the total mixed ration (TMR). Table 1 presents the composition and nutritional levels of the basal diet. Net energy for maintenance and growth (NEmf) was calculated according to the “Feeding Standard for Meat Sheep” (NY/T 816-2021).

### 2.2. Measurement of Growth Performance

The sheep were weighed before morning feeding on days 0 and 30. Average daily gain (ADG) was calculated as the difference between final and initial body weight (BW) divided by the 30-day experimental period. Daily feed intake was recorded to determine average daily dry matter intake (ADMI) and feed-to-gain ratio (F/G).

### 2.3. Sample Collection

During the collection period (days 24–30), fresh fecal grab samples (approximately 200 g each) were collected from each sheep at 4 h intervals (00:00, 04:00, 08:00, 12:00, 16:00, and 20:00 h) to account for diurnal variation in fecal excretion. Feed samples were collected concurrently. The 6 daily fecal grab samples from each sheep were pooled by day, and the 7 daily pools were then thoroughly mixed to obtain one composite sample per animal for the entire collection period. A 10% aliquot of the composite sample was retained and stored at −20 °C until nutrient analysis. Before analysis, samples were dried at 65 °C for 72 h, ground, and passed through a 40-mesh sieve (0.425 mm).

On day 30 of the trial, blood samples were collected via jugular venipuncture. After standing at room temperature for 30 min, the samples were centrifuged at 3000× *g* for 10 min at 4 °C, and the resulting serum was aliquoted and stored at −20 °C until analysis.

On day 30, rumen fluid samples were collected from sheep approximately 6 h after the morning feeding using an oral stomach tube. To minimize saliva contamination, the first 2 aliquots were discarded. The remaining rumen fluid was filtered through 4 layers of surgical gauze, and pH was measured immediately. A portion of the filtrate was stored at −20 °C for rumen fermentation analysis, and another portion was aliquoted into sterile cryovials and stored at −80 °C for microbial analysis. For 16S rRNA gene sequencing, one sheep per replicate pen was randomly selected as the representative (*n* = 4 per group), with the replicate pen as the experimental unit for microbial community analysis. For rumen fermentation parameters, samples from all 12 sheep per group were analyzed, with individual sheep as the experimental unit.

### 2.4. Determination of Nutrient Levels in Feed and Feces

Samples were dried at 65 °C for 72 h, ground, and passed through a 40-mesh sieve (0.425 mm). DM, crude protein (CP), neutral detergent fiber (NDF), acid detergent fiber (ADF), calcium, and total phosphorus contents in feed and feces were measured as described by Chen et al. [20]. The feed and fecal nutrient analyses were conducted between January and February 2025. Ash content was determined using a muffle furnace (Model SX2-12-10, Shanghai Yuejin Medical Instruments Co., Ltd., Shanghai, China) following GB/T 6438–2007 [21]. Ether extract (EE) was analyzed using a Soxhlet extractor (250 mL, 29/32 joint, Huaguang Glass, Wuxi, China) in accordance with GB/T 6433–2006 [22]. These standards were the applicable versions during the analytical period. The apparent digestibility of nutrients was determined using the acid-insoluble ash (AIA) method, which employs AIA concentration as an internal indigestible marker [23].

### 2.5. Rumen Fermentation Parameter Analysis

VFAs were quantified using an Agilent 7820A gas chromatograph (Agilent Technologies, Santa Clara, CA, USA) equipped with a capillary column (Agilent Technologies, Santa Clara, CA, USA; 30 m × 0.25 mm × 0.33 μm). Sample preparation and parameters followed the method of Xiao et al. [24]. Ammonia nitrogen (NH_3_-N) concentration was analyzed colorimetrically using a spectrophotometer (UV-2450, Shimadzu, Tokyo, Japan) [25]. Rumen MCP concentration was determined colorimetrically with a commercial enzyme-linked detection system (Suzhou Geruisi Biotechnology Co., Ltd., Suzhou, China; Cat. No. G0418W).

### 2.6. Serum Parameter Analysis

Blood urea nitrogen (BUN), total protein (TP), albumin (ALB), and glucose (GLU) were analyzed using an automatic biochemical analyzer (BS-2800M, Mindray, Shenzhen, China). Serum antioxidant indicators, including catalase (CAT), superoxide dismutase (SOD), glutathione peroxidase (GSH-Px), and malondialdehyde (MDA), were measured using commercial assay kits (Nanjing Jiancheng Bioengineering Institute, Nanjing, China).

### 2.7. 16S rRNA Gene Sequencing

For rumen microbial analysis, one sheep was randomly selected from each of the four replicate pens in each treatment group, resulting in four biological replicates per treatment. Rumen fluid samples collected from these sheep were used for 16S rRNA gene sequencing. Because dietary treatment was assigned at the pen level, each pen was considered an independent experimental unit.

Rumen microbial DNA was extracted using a commercial genomic DNA extraction kit (EC801; TransGen Biotech Co., Ltd., Beijing, China) according to the manufacturer’s instructions. PCR amplification and sequencing were performed by Novogene Co., Ltd. (Beijing, China). The V3–V4 region of the 16S rRNA gene was amplified using primers 341F (5′-CCTAYGGGRBGCASCAG-3′) and 806R (5′-GGACTACNNGGGTATCTAAT-3′), as described by Hjelmsø et al. [26]. PCR amplification consisted of initial denaturation at 98 °C for 1 min, followed by 30 cycles at 98 °C for 10 s, 50 °C for 30 s, and 72 °C for 30 s, with a final extension at 72 °C for 5 min. Amplicons were sequenced on an Illumina MiSeq platform (Illumina, Inc., San Diego, CA, USA) using a 2 × 300 bp paired-end sequencing strategy. Raw reads were merged and quality-filtered, and operational taxonomic units (OTUs) were clustered at 97% similarity using USEARCH (v11.0.667). Taxonomic annotation was performed using the RDP Classifier against the SILVA database (release 138.1), and downstream analyses were conducted in QIIME 2 (version 2022.2).

Alpha diversity indices were calculated to evaluate the richness and diversity of the rumen microbial community. Beta diversity was assessed by principal coordinate analysis (PCoA). Differential microbial taxa among treatments were identified by linear discriminant analysis effect size (LEfSe, v1.1.01). Functional profiles of the rumen microbial community were predicted using Tax4Fun (v0.3.1) based on the Kyoto Encyclopedia of Genes and Genomes (KEGG) database. Spearman correlation analysis was further performed to assess the relationships between key rumen microbial taxa, predicted microbial functions, and major phenotypic parameters.

### 2.8. Statistical Analysis

Data are presented as the mean ± standard error of the mean (SEM), except for the alpha-diversity indices, which are presented as the mean ± standard deviation (SD). Statistical analyses were performed using SPSS 26.0 software (SPSS Inc., Chicago, IL, USA). Growth performance, serum indices, nutrient digestibility, rumen fermentation parameters, and the relative abundances of major microbial taxa and predicted microbial functions were analyzed by one-way analysis of variance (ANOVA), followed by Tukey’s multiple-comparison test for multiple comparisons. In addition, linear and quadratic dose–response effects were evaluated using orthogonal polynomial contrasts based on the actual unequally spaced puerarin supplementation levels of 0, 50, 100, 200, 400, and 800 mg/kg DM. Alpha-diversity indices were analyzed using the Kruskal–Wallis test followed by Dunn’s post hoc test, with *p* values adjusted using the Benjamini–Hochberg false discovery rate procedure. Alpha-diversity data are presented as the mean ± SD. Differential microbial taxa among treatments were identified by LEfSe analysis. For LEfSe analysis, the significance threshold for the Kruskal–Wallis test was set at *p* < 0.05, and the logarithmic linear discriminant analysis score threshold was set at 2.0. To evaluate whether the numerical differences in initial BW affected the final BW results, final BW was additionally analyzed using analysis of covariance (ANCOVA), with dietary treatment as the fixed effect and initial BW as the covariate. Homogeneity of regression slopes was assessed using the dietary treatment × initial BW interaction. When the interaction was not significant, it was removed from the final model. Bonferroni adjustment was applied to pairwise comparisons of the estimated marginal means. Differences were considered statistically significant when *p* < 0.05.

## 3. Results

### 3.1. Growth Performance

The growth performance results are presented in Table 2. After 30 d of feeding, puerarin supplementation significantly affected ADG (ANOVA, *p* = 0.031). Compared with the control group, ADG was significantly increased in the 50, 100, and 400 mg/kg puerarin groups, with the highest value observed in the 100 mg/kg group. As the puerarin supplementation level increased, ADG showed a quadratic response (*p*-quadratic = 0.039). Initial BW, final BW, ADMI, and F/G were not significantly affected by treatment in the one-way ANOVA and showed no significant linear or quadratic responses (*p* > 0.05). The dietary treatment × initial BW interaction was not significant (*p* = 0.649), indicating that the homogeneity-of-regression-slopes assumption was satisfied. After adjustment for initial BW, dietary treatment significantly affected final BW (*p* = 0.038), and the adjusted final BW was greater in the 100 mg/kg group than in the control group (*p* = 0.039; Table A1).

### 3.2. Apparent Nutrient Digestibility

The apparent nutrient digestibility results are shown in Table 3. Among the measured indices, only ADF digestibility was significantly affected by puerarin supplementation (*p* = 0.015). The 100 mg/kg group showed the highest ADF digestibility, which was significantly greater than that of the control group. As the puerarin supplementation level increased, ADF digestibility showed a quadratic response (*p*-quadratic = 0.048). No significant treatment, linear, or quadratic effects were observed for the digestibility of DM, CP, EE, or NDF (*p* > 0.05).

### 3.3. Rumen Fermentation

As shown in Table 4, puerarin supplementation significantly affected several rumen fermentation parameters. Compared with the control group, MCP concentration was significantly increased in the 100 and 400 mg/kg groups (ANOVA, *p* = 0.043), and MCP showed a quadratic response as the puerarin level increased (*p*-quadratic = 0.025). Propionate concentration was significantly increased in the 50 and 100 mg/kg groups (*p* = 0.008) and showed a significant linear dose–response component (*p*-linear = 0.004). A/P (acetate-to-propionate ratio) was significantly decreased in the 100 mg/kg group (ANOVA, *p* = 0.035), but showed no significant linear or quadratic response (*p* > 0.05).

Although NH_3_-N concentration did not differ significantly among treatments (ANOVA, *p* = 0.419), it decreased linearly with increasing puerarin supplementation (*p*-linear = 0.041). Acetate and total VFAs were significantly decreased in the 800 mg/kg group (ANOVA, *p* = 0.008 and 0.009, respectively). Acetate and total VFAs exhibited both linear and quadratic dose–response components (acetate: *p*-linear = 0.004 and *p*-quadratic = 0.023; total VFA: *p*-linear = 0.003 and *p*-quadratic = 0.018). No significant treatment, linear, or quadratic effects were observed for butyrate concentration (*p* > 0.05).

### 3.4. Serum Biochemical and Antioxidant Indices

Serum biochemical and antioxidant indices are summarized in Table 5. Puerarin supplementation significantly affected serum MDA, ALB, and GLU concentrations. Compared with the control group, serum MDA concentration was significantly decreased in the 100 mg/kg group but significantly increased in the 800 mg/kg group (*p* < 0.001). MDA showed a significant linear dose–response component across the tested supplementation range (*p*-linear < 0.001), whereas the quadratic response was not significant (*p*-quadratic = 0.536).

Serum ALB concentration was significantly increased in the 100, 200, and 400 mg/kg groups (ANOVA, *p* = 0.044) and showed a quadratic response to increasing puerarin supplementation (*p*-quadratic = 0.005). Serum GLU concentration was significantly increased in the 100 and 200 mg/kg groups (ANOVA, *p* = 0.004) and also exhibited a quadratic response (*p*-quadratic = 0.007). No significant treatment, linear, or quadratic effects were observed for GSH-Px, SOD, CAT, BUN, or TP (*p* > 0.05).

### 3.5. Composition of Rumen Microbial Community

**Figure 1:**
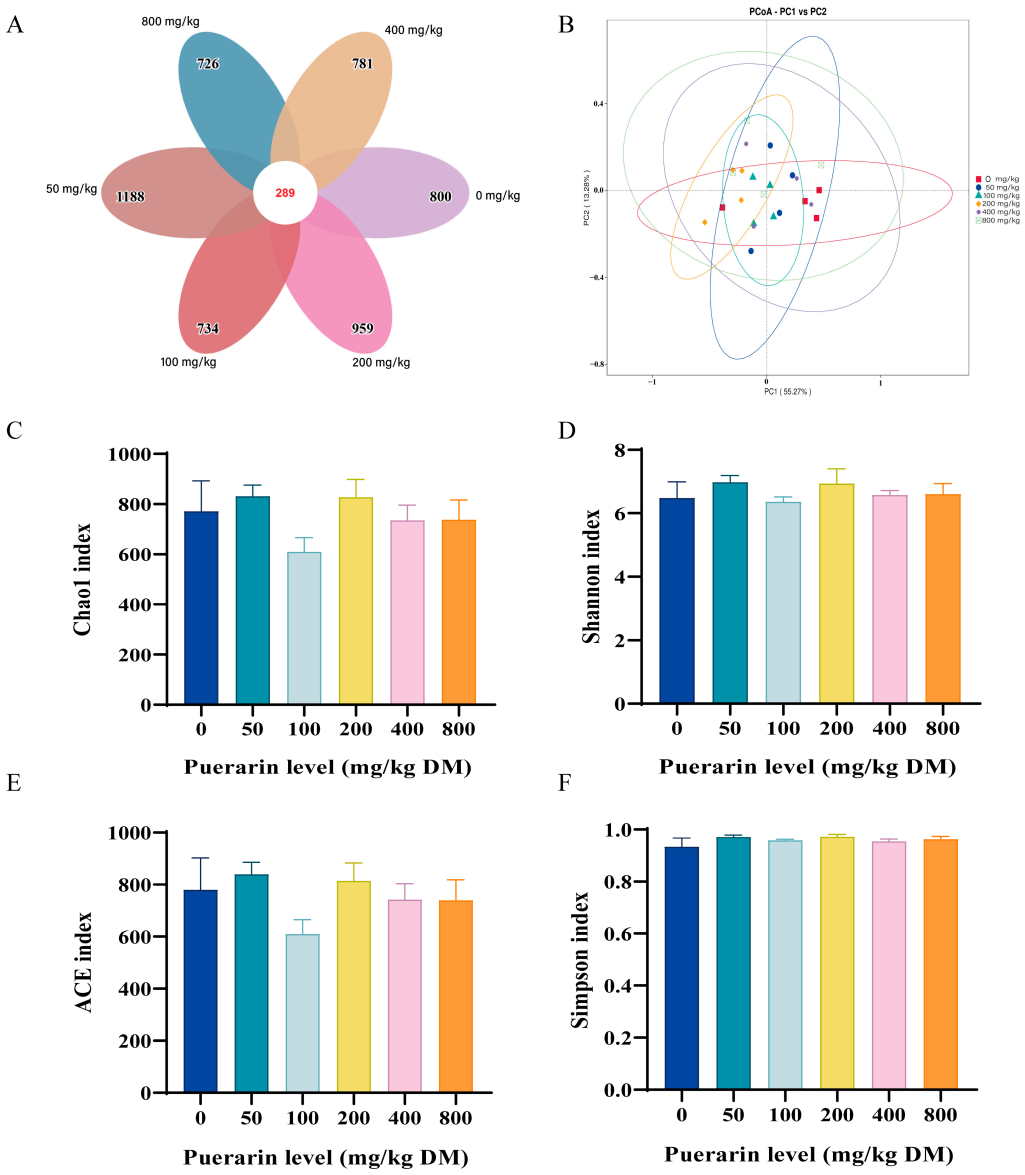
Effects of dietary puerarin supplementation on rumen microbial diversity in Hu sheep. (**A**) Flower diagram showing the shared and unique operational taxonomic units (OTUs) among the six treatment groups. (**B**) Principal coordinate analysis (PCoA) of the rumen bacterial communities based on weighted UniFrac distances; The colored ellipses represent the 95% confidence intervals for each treatment group. (**C**–**F**) Alpha-diversity indices of the rumen microbial community, including Chao1 (**C**), Shannon (**D**), ACE (**E**), and Simpson (**F**). The dietary puerarin supplementation levels were 0, 50, 100, 200, 400, and 800 mg/kg on a dry matter basis. Each treatment group included four biological replicates (*n* = 4). Data in panels (**C**–**F**) are presented as mean ± SD. Differences among treatments were analyzed using the Kruskal–Wallis test followed by Dunn’s post hoc test with Benjamini–Hochberg false discovery rate correction. No significant differences were detected among treatments for any alpha-diversity index (*p* > 0.05).

A total of 289 OTUs were shared among all six treatment groups, while each group also contained unique OTUs (Figure 1A). The PCoA plot showed substantial overlap among groups, suggesting no obvious differences in overall beta diversity (Figure 1B). Similarly, the Chao1, Shannon, ACE, and Simpson indices did not differ significantly among treatments (*p* > 0.05; Figure 1C–F).

**Figure 2:**
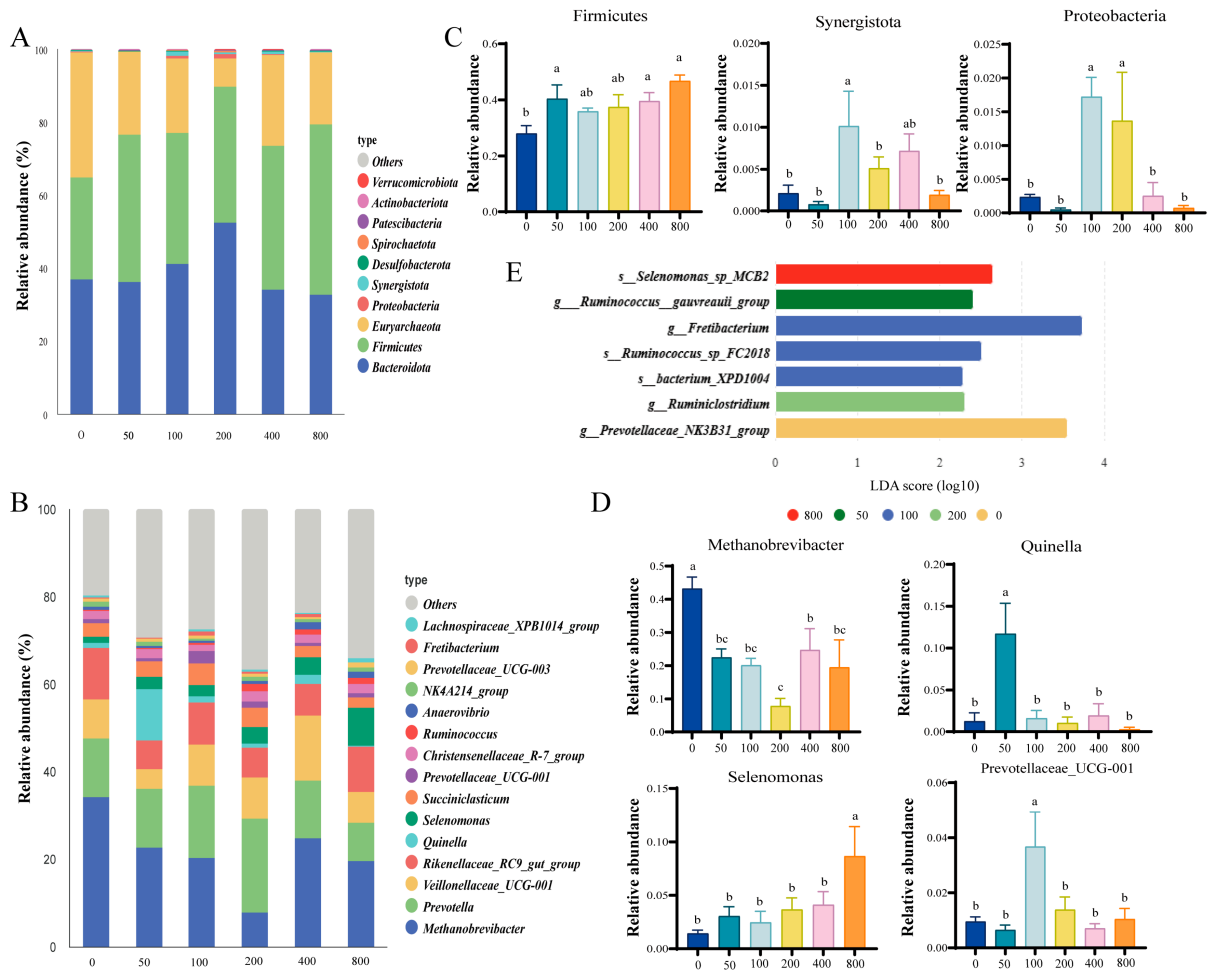
Effects of dietary puerarin supplementation on rumen microbial composition in Hu sheep. (**A**) Relative abundance of the dominant microbial taxa at the phylum level. (**B**) Relative abundance of the dominant microbial taxa at the genus level. (**C**) Relative abundances of Firmicutes, Synergistota, and Proteobacteria among treatments. (**D**) Relative abundances of *Methanobrevibacter*, *Quinella*, *Selenomonas*, and Prevotellaceae_UCG-001 among treatments. (**E**) Linear discriminant analysis effect size (LEfSe) analysis showing the discriminative microbial taxa enriched in the different treatment groups. The numbers 0, 50, 100, 200, 400, and 800 on the x-axis represent dietary puerarin supplementation levels in mg/kg DM. Data in panels (**C**,**D**) are presented as mean ± SEM (*n* = 4 per treatment). Different lowercase letters indicate significant differences among treatments (*p* < 0.05). Only treatment groups with taxa meeting the LEfSe screening criteria are shown in panel (**E**). Only taxa with an LDA score > 2.0 and *p* < 0.05 are shown.

At the phylum level, Bacteroidota, Euryarchaeota, and Firmicutes were the dominant taxa in the rumen fluid of Hu sheep, accounting for more than 98% of the total sequences (Figure 2A). Compared with the control group, Firmicutes was significantly increased in the 50, 400, and 800 mg/kg groups, whereas Proteobacteria was significantly increased in the 100 and 200 mg/kg groups (*p* < 0.05). In addition, Synergistota was significantly enriched in the 100 mg/kg group (Figure 2C; *p* < 0.05).

At the genus level, *Methanobrevibacter* and *Prevotella* were among the dominant genera across treatments (Figure 2B). Compared with the control group, the relative abundance of *Methanobrevibacter* was significantly reduced in all puerarin-supplemented groups (*p* = 0.007). *Quinella* was significantly increased in the 50 mg/kg group (*p* = 0.002), Prevotellaceae_UCG-001 was significantly increased in the 100 mg/kg group (*p* = 0.007), and *Selenomonas* was significantly increased in the 800 mg/kg group (Figure 2D; *p* = 0.035).

LEfSe analysis further identified distinct bacterial biomarkers among treatments (Figure 2E). The control group was characterized by enrichment of Prevotellaceae_NK3B31_group, whereas the 50 mg/kg group was enriched in Ruminococcus_gauvreauii_group. The 100 mg/kg group was characterized by enrichment of *Fretibacterium*, *Ruminococcus*_sp_FC2018, and bacterium_XPD1004. In the 200 mg/kg group, *Ruminiclostridium* was identified as a representative biomarker, whereas the 800 mg/kg group was enriched in *Selenomonas*_sp_MCB2.

### 3.6. Predicted Microbial Functions

**Figure 3:**
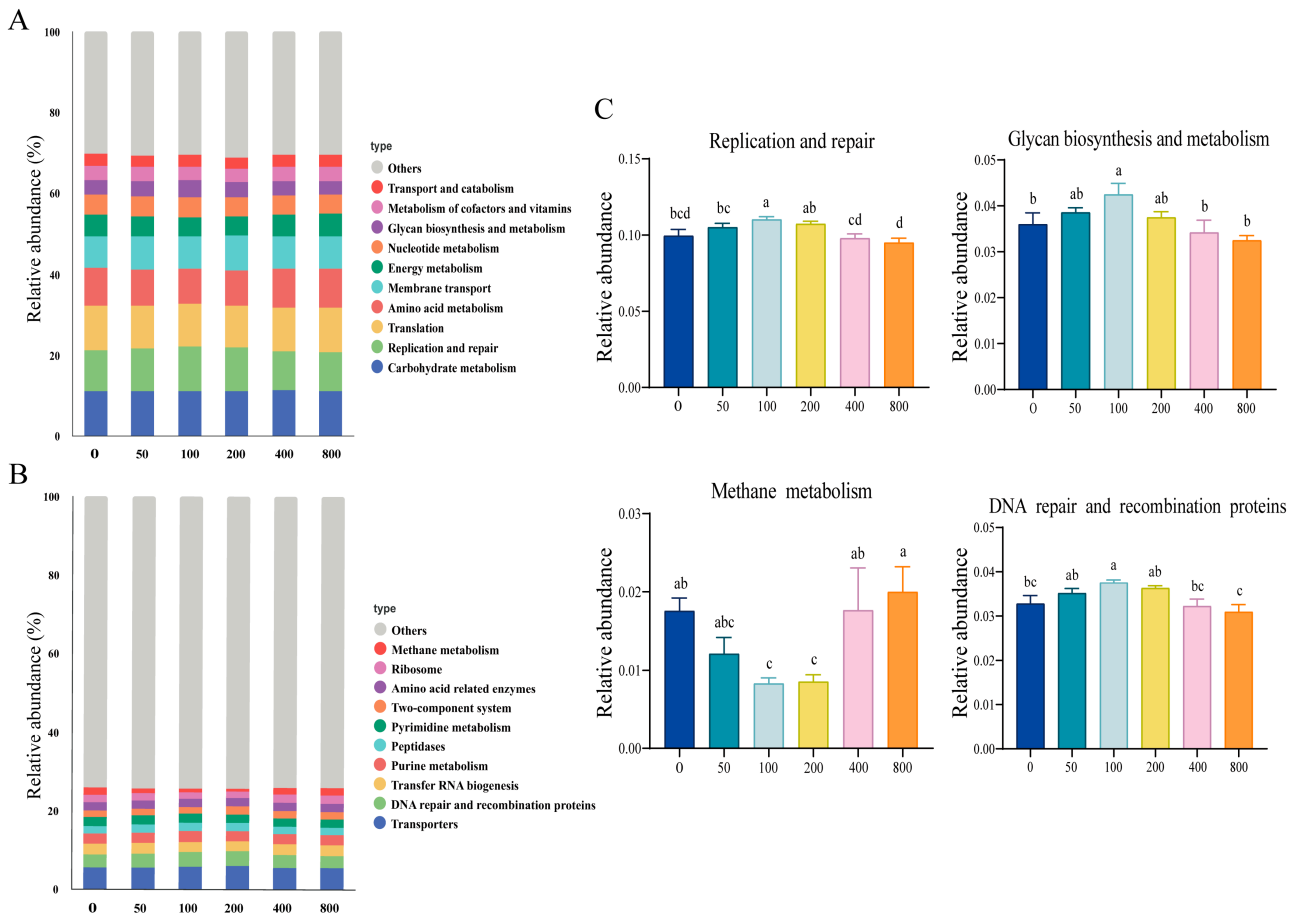
Effects of dietary puerarin supplementation on the predicted functional profiles of the rumen microbial community in Hu sheep. (**A**) Relative abundance of the major Tax4Fun-predicted microbial functions at Kyoto Encyclopedia of Genes and Genomes (KEGG) level 2. (**B**) Relative abundance of the major predicted microbial functions at KEGG level 3. (**C**) Relative abundances of selected predicted microbial functions that differed among treatments, including replication and repair, glycan biosynthesis and metabolism, methane metabolism, and DNA repair and recombination proteins. The numbers 0, 50, 100, 200, 400, and 800 on the x-axis represent dietary puerarin supplementation levels in mg/kg DM. Data in panel C are presented as mean ± SEM (*n* = 4 per treatment). Different lowercase letters indicate significant differences among treatments (*p* < 0.05).

As shown in Figure 3, Tax4Fun-based functional prediction suggested that puerarin selectively affected the predicted functional profile of the rumen microbiota. At level 2, pathways related to replication and repair and glycan biosynthesis and metabolism were significantly affected among treatments. At level 3, methane metabolism was significantly decreased, whereas DNA repair and recombination proteins were significantly increased. These results suggest that puerarin may influence specific microbial functional potentials associated with fermentation pattern and microbial adaptation.

### 3.7. Correlation Analysis

**Figure 4:**
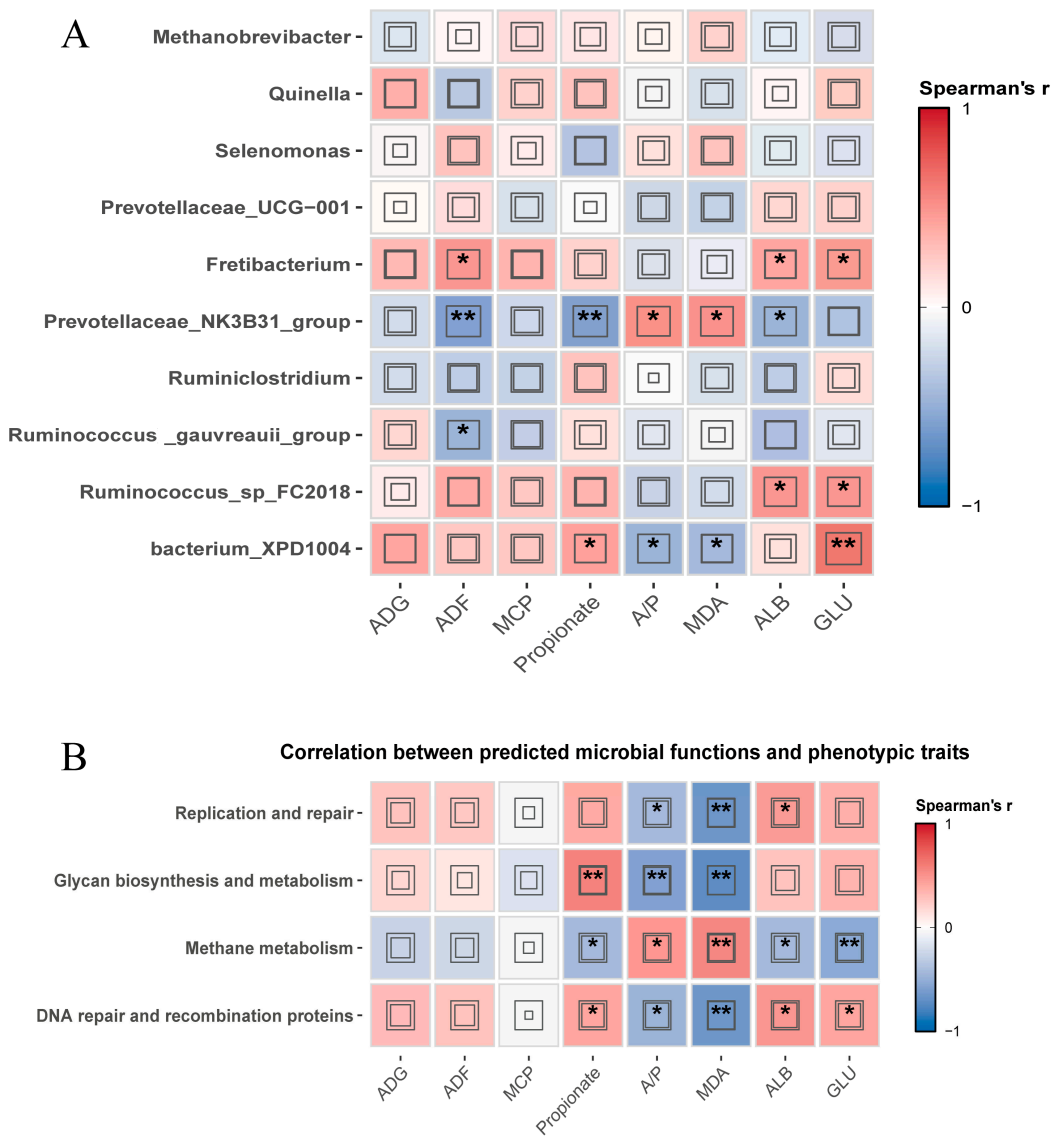
Spearman correlation analysis among differential rumen microbes, predicted microbial functions, and phenotypic traits in Hu sheep. (**A**) Correlation heatmap between key differential rumen microbial taxa and phenotypic traits. (**B**) Correlation heatmap between selected predicted microbial functions and phenotypic traits. The color indicates the direction and strength of the correlation, with red representing positive correlations and blue representing negative correlations. The size of the inner square is proportional to the absolute value of Spearman’s correlation coefficient, with larger squares indicating stronger correlations. Asterisks indicate significant correlations (* *p* < 0.05, ** *p* < 0.01). ADF = acid detergent fiber digestibility; MCP = microbial crude protein; A/P = acetate-to-propionate ratio; MDA = malondialdehyde; ALB = albumin; GLU = glucose.

Spearman correlation analysis revealed selective associations between puerarin-responsive rumen microbes and host phenotypic traits (Figure 4A). Specifically, *Fretibacterium* was positively correlated with ADF digestibility, ALB, and GLU (*p* < 0.05). In contrast, Prevotellaceae_NK3B31_group showed negative correlations with ADF digestibility, propionate, and ALB, but positive correlations with the A/P and MDA (*p* < 0.05 or *p* < 0.01). In addition, Ruminococcus_gauvreauii_group was negatively correlated with ADF digestibility (*p* < 0.05), whereas *Ruminococcus*_sp_FC2018 was positively correlated with ALB and GLU (*p* < 0.05). Notably, bacterium_XPD1004 was positively correlated with propionate and GLU, but negatively correlated with the A/P and MDA (*p* < 0.05 or *p* < 0.01). At the functional level (Figure 4B), the pathway “replication and repair” was negatively correlated with A/P and MDA, but positively correlated with ALB (*p* < 0.05). Glycan biosynthesis and metabolism showed a positive correlation with propionate (*p* < 0.01) and negative correlations with A/P and MDA (*p* < 0.01). In contrast, methane metabolism was negatively correlated with propionate, ALB, and GLU, but positively correlated with A/P and MDA (*p* < 0.05 or *p* < 0.01). Furthermore, the pathway DNA repair and recombination proteins was positively correlated with propionate, ALB, and GLU, but negatively correlated with A/P and MDA (*p* < 0.05 or *p* < 0.01). These results indicate that puerarin-responsive microbial taxa and predicted microbial functions were closely associated with a more favorable rumen fermentation pattern and improved metabolic and antioxidant status in Hu sheep.

## 4. Discussion

In meat sheep production, the economic returns of fattening lambs largely depend on growth performance and feed utilization efficiency. ADG serves as a critical indicator of farming profitability [27,28]. Puerarin has attracted increasing attention as a plant-derived bioactive compound with potential applications in animal production. Previous studies in broilers and piglets have shown that puerarin supplementation can improve growth performance, feed efficiency, and physiological status [29,30]. In the present study, dietary puerarin supplementation significantly increased ADG, with the greatest value observed in the 100 mg/kg group. In addition, ADF digestibility was significantly improved in the 100 mg/kg group. The concurrent improvement in ADF digestibility and ADG at the 100 mg/kg dose suggests that enhanced fiber utilization may have contributed to the observed growth response. However, because the formal feeding period was limited to 30 days, the observed improvement in ADG should be interpreted as a short-term growth response under the present experimental conditions. Longer feeding trials covering a greater proportion of the fattening period are required to determine whether this response can be sustained over time.

In ruminants, growth performance is closely linked to rumen fermentation efficiency, because ruminal microorganisms degrade dietary substrates into VFAs and synthesize MCP, which are major contributors to host energy and amino acid supply [31,32]. Among VFAs, propionate is particularly important because it serves as the principal glucogenic precursor in ruminants [33,34]. Consequently, dynamic changes in the composition of the rumen microbiota directly affect fermentation patterns, tightly linking the structure of the rumen microbial community to its production traits, such as ADG and feed efficiency [35]. In the present study, puerarin supplementation, particularly at 100 mg/kg, increased propionate and MCP concentrations, decreased A/P, and increased serum GLU concentration. These changes indicate that puerarin shifted rumen fermentation toward a more favorable pattern, which may have enhanced nutrient conversion efficiency and energy availability. Consistent with this interpretation, serum GLU concentration was significantly elevated in the 100 and 200 mg/kg groups. Because glucose supply in ruminants depends largely on hepatic gluconeogenesis from propionate, the increase in serum GLU further supports the view that puerarin improved the efficiency of energy metabolism through modulation of rumen fermentation [36,37]. Notably, this improvement in energy availability occurred without a corresponding increase in ADMI, suggesting that puerarin enhanced energy extraction efficiency rather than simply promoting greater consumption.

In addition to effects on fermentation, puerarin supplementation altered several serum biochemical and antioxidant indices. Serum ALB concentration was significantly increased in the 100, 200, and 400 mg/kg groups, suggesting an improvement in protein nutritional status and metabolic homeostasis. ALB is not only an indicator of protein synthesis capacity but also an important marker reflecting the overall metabolic condition of the animal [38,39]. Meanwhile, serum MDA concentration was significantly reduced in the 100 mg/kg group but markedly increased in the 800 mg/kg group. This pattern suggests that an appropriate level of puerarin may alleviate oxidative stress, whereas excessive supplementation may impose a pro-oxidative burden. Similar dose-dependent responses of plant-derived bioactive compounds have been reported previously, indicating that moderate supplementation can be beneficial, but excessive doses may reduce or even reverse the positive effects [40,41,42,43]. Therefore, the present findings suggest that 100 mg/kg puerarin may represent a more suitable supplementation level for improving both metabolic status and antioxidant balance in Hu sheep.

The 16S rRNA sequencing results further indicated that dietary puerarin supplementation had limited effects on overall rumen microbial diversity. Alpha-diversity indices did not differ significantly among treatments, and the PCoA plot showed substantial overlap among treatment groups, suggesting no obvious change in overall beta diversity. These findings indicate that the effects of puerarin on rumen fermentation were more likely associated with changes in specific microbial taxa than with broad restructuring of the rumen microbial community.

At the phylum level, Bacteroidota, Firmicutes, and Euryarchaeota remained the dominant taxa across all groups, which is consistent with the typical rumen bacterial composition reported in sheep and other ruminants [44,45]. Compared with the control group, Firmicutes was significantly increased in the 50, 400, and 800 mg/kg groups, whereas Proteobacteria was significantly increased in the 100 and 200 mg/kg groups, and Synergistota was enriched in the 100 mg/kg group. Among the phyla affected by puerarin, Firmicutes have been widely linked to fiber degradation and feed utilization, with several Firmicutes-affiliated genera reported to play pivotal roles in NDF and ADF degradation in Hu sheep [26], whereas Proteobacteria and Synergistota may participate in specific substrate transformation processes in the rumen [46,47]. These phylum-level shifts suggest that puerarin did not broadly disrupt the dominant rumen microbial composition, but was associated with changes in microbial groups related to fiber degradation, substrate transformation, and fermentation pattern. Together with the increased ADF digestibility, higher MCP and propionate concentrations, lower A/P, and greater ADG observed in the present study, these findings suggest that puerarin may enhance nutrient utilization and growth performance in Hu sheep by improving rumen fermentation and influencing specific components of the rumen microbiota.

At the genus level, puerarin supplementation significantly reduced the relative abundance of *Methanobrevibacter* in all treatment groups. *Methanobrevibacter* is one of the major methanogenic archaea in the rumen and is directly involved in methane formation through hydrogen utilization [48,49]. A reduction in this genus may imply that less metabolic hydrogen was directed toward methanogenesis, potentially favoring alternative electron-sinking fermentation pathways, including propionate formation [50]. This interpretation is consistent with the observed increase in propionate concentration and the lower A/P in the present study. In addition, *Quinella* was significantly increased in the 50 mg/kg group, Prevotellaceae_UCG-001 was significantly increased in the 100 mg/kg group, and *Selenomonas* was significantly increased in the 800 mg/kg group. *Quinella* and *Selenomonas* have frequently been associated with carbohydrate fermentation and propionate-related pathways in the rumen [51,52,53], whereas members of Prevotellaceae are widely involved in the degradation of dietary substrates and fermentation product formation [54]. Therefore, the enrichment of these taxa further supports the idea that puerarin may have influenced specific components of the rumen microbiota toward a fermentation pattern more favorable for host nutrient utilization.

LEfSe analysis provided further evidence that different puerarin levels enriched different microbial biomarkers. The control group was characterized by Prevotellaceae_NK3B31_group, whereas the 50 mg/kg group was enriched in Ruminococcus_gauvreauii_group. The 100 mg/kg group was characterized by enrichment of *Fretibacterium*, *Ruminococcus*_sp_FC2018, and bacterium_XPD1004, while the 200 mg/kg group was characterized by *Ruminiclostridium* and the 800 mg/kg group by *Selenomonas*_sp_MCB2. This pattern indicates that puerarin exerted dose-dependent effects on specific rumen microbial taxa. Notably, the unique enrichment of *Ruminococcus*-related taxa at the 100 mg/kg supplementation level is of particular interest. Even though the total phylum-level abundance of Firmicutes did not shift significantly at this specific dose, the targeted proliferation of these core fiber-degrading genera likely served as the primary biological driver for the improved ADF digestibility observed in the 100 mg/kg group [55]. These findings suggest that the beneficial effects of puerarin on performance and fermentation may be associated with changes in bacterial groups involved in fiber utilization and favorable fermentation pathways.

Tax4Fun-based functional prediction further suggested that puerarin selectively altered specific microbial functions rather than broadly reshaping the overall functional profile of the rumen microbiota. At level 2, replication and repair and glycan biosynthesis and metabolism were significantly affected among treatments, with both showing the highest relative abundance in the 100 mg/kg group. At level 3, methane metabolism was significantly decreased, whereas DNA repair and recombination proteins showed a significant increase. The reduced abundance of *Methanobrevibacter*, together with the decline in predicted methane metabolism, suggests that puerarin may suppress methanogenesis-related functional potential in the rumen microbiota [56]. By contrast, the enrichment of replication- and repair-related functions may reflect altered microbial adaptation and genomic maintenance under puerarin supplementation [57]. The increase in glycan biosynthesis and metabolism further suggests changes in carbohydrate-related microbial functions that may be associated with improved substrate utilization and fermentation efficiency [58]. Because Tax4Fun is based on 16S-derived function prediction, these results should be regarded as putative functional shifts rather than direct measurements of metabolic activity. Nevertheless, they provide complementary evidence supporting the observed improvements in rumen fermentation.

Rumen microorganisms have been recognized as key regulators of nutrient acquisition and health status in ruminants [27,59]. Changes in rumen microbial composition can influence host physiological traits by affecting feed degradation, fermentation pattern, and nutrient utilization efficiency [59]. In the present study, the correlation analyses further supported the association between puerarin-responsive rumen microbes and host phenotypic traits. Specifically, *Fretibacterium* abundance was positively associated with ADF digestibility, ALB, and GLU, suggesting that this taxon may contribute to fiber degradation and host metabolic status, or alternatively, that its proliferation is favored by the same environmental conditions that promote these phenotypic traits. In addition, *Ruminococcus*_sp_FC2018 and bacterium_XPD1004 were positively associated with favorable fermentation and metabolic traits, including higher propionate, ALB, or GLU, and lower A/P or MDA. These correlations suggest that puerarin-induced shifts in the rumen microbiota were associated with taxa linked to phenotypic traits indicative of enhanced fermentation efficiency and metabolic status.

At the functional level, the predicted microbial functions showed a similar pattern of association with host traits. Glycan biosynthesis and metabolism and DNA repair and recombination proteins were generally associated with higher propionate, ALB, and GLU, whereas methane metabolism showed the opposite tendency and was positively associated with A/P and MDA. Together, these findings suggest that puerarin may improve rumen fermentation efficiency and host metabolic status by influencing specific responsive microbial taxa and the predicted functional potential of the rumen microbiota. A limitation of the present study is that rumen microbial sequencing was conducted using one sheep from each replicate pen, resulting in four biological replicates per treatment. Although this design ensured representation of all independent pens and avoided pseudoreplication, it may not have fully captured within-pen variation and may have limited the statistical power to detect subtle treatment-associated differences, particularly in low-abundance microbial taxa. Future studies involving a larger number of independent experimental units are warranted to validate the observed microbial responses.

## 5. Conclusions

Dietary puerarin supplementation improved short-term growth performance, rumen fermentation, nutrient utilization, and metabolic status in Hu sheep, with 100 mg/kg showing the most favorable overall response. Specifically, puerarin increased ADG, improved ADF digestibility, enhanced ruminal MCP and propionate concentrations, decreased A/P, increased serum ALB and GLU concentrations, and decreased serum MDA concentration at appropriate supplementation levels. These beneficial effects were accompanied by alterations in certain rumen microbial taxa and predicted microbial functions. Collectively, the results suggest that puerarin may enhance nutrient utilization and growth performance in Hu sheep, at least in part, through improving rumen fermentation and affecting specific components of the rumen microbiota.

## Figures and Tables

**Table 1:** Ingredient composition and nutrient levels of the basal diet (% of DM).

Ingredients	Content	Nutrient Levels ^1^	Content
Peanut vine	60.00	NE_mf_, MJ/kg	6.15
Corn	21.6	DM	85.80
Wheat bran	4.40	Ash	9.61
Soybean meal	9.72	CP	15.09
NaHCO_3_	0.92	NDF	32.70
NaCl	0.52	ADF	20.81
Na_2_HPO_4_	0.84	Calcium	0.98
Premix ^2^	2.00	Phosphorus	0.50
Total	100		

^1^ DM = dry matter; NE_mf_ = net energy for maintenance and growth; CP = crude protein; NDF = neutral detergent fiber; ADF = acid detergent fiber. ^2^ One kilogram of premix contained the following: vitamin A 250,000 IU, vitamin D_3_, 100,000 IU, calcium 150,000 mg, phosphorus 50,000 mg, vitamin E 3000 IU, Fe 1000 mg, Mn 3000 mg, Zn 3000 mg, Cu 500 mg, I 8 mg, Se 10 mg, Co 10 mg. NEmf was a calculated value, while the others were measured values.

**Table 2:** Effects of puerarin on the growth performance of Hu sheep.

Items	Puerarin Levels, mg/kg						SEM	*p*-Value		
	0	50	100	200	400	800		ANOVA	Linear	Quadratic
Initial BW ^1^, kg	19.625	21.850	21.833	21.142	21.625	21.083	0.640	0.936	0.892	0.604
Final BW, kg	24.025	27.392	27.933	26.400	27.200	25.875	0.675	0.635	0.996	0.344
ADG ^2^, kg/d	0.147 ^c^	0.185 ^ab^	0.203 ^a^	0.175 ^abc^	0.186 ^ab^	0.160 ^bc^	0.006	0.031	0.479	0.039
ADMI ^3^, kg/d	0.933	1.128	1.122	1.068	1.109	1.017	0.026	0.171	0.852	0.103
F/G ^4^	6.393	6.175	5.585	6.087	5.988	6.548	0.179	0.772	0.528	0.372

^1^ BW = body weight; ^2^ ADG = average daily gain; ^3^ ADMI = average dry matter intake; ^4^ F/G = feed-to-gain ratio. ^a–c^ Means in a row not sharing a common letter are significantly different (*p* < 0.05).

**Table 3:** Effects of dietary puerarin supplementation on apparent nutrient digestibility in Hu sheep (%).

Items ^1^	Puerarin Levels, mg/kg						SEM	*p*-Value		
	0	50	100	200	400	800		ANOVA	Linear	Quadratic
DM	77.514	82.350	81.739	80.778	79.097	78.486	0.685	0.291	0.353	0.530
CP	75.185	73.068	74.493	71.503	72.034	70.096	0.823	0.563	0.161	0.962
EE	76.653	76.902	78.724	76.230	75.415	73.001	0.871	0.668	0.139	0.807
NDF	50.909	50.444	56.143	51.838	50.795	53.170	1.415	0.881	0.879	0.913
ADF	39.605 ^bc^	37.193 ^c^	50.251 ^a^	43.159 ^abc^	45.380 ^ab^	41.679 ^bc^	1.244	0.015	0.752	0.048

^1^ DM = dry matter; CP = crude protein; EE = ether extract; NDF = neutral detergent fiber; ADF = acid detergent fiber. ^a–c^ Means in a row not sharing a common letter are significantly different (*p* < 0.05).

**Table 4:** Effects of puerarin on rumen fermentation in Hu sheep.

Items	Puerarin Levels, mg/kg						SEM	*p*-Value		
	0	50	100	200	400	800		ANOVA	Linear	Quadratic
pH	7.122	7.138	7.208	7.093	7.095	7.290	0.024	0.112	0.059	0.078
MCP ^1^, μg/mL	593.452 ^b^	700.235 ^ab^	890.191 ^a^	760.670 ^ab^	880.780 ^a^	785.633 ^ab^	31.975	0.043	0.154	0.025
NH_3_-N, mg/dL	7.995	7.853	7.573	7.587	7.396	6.956	0.146	0.419	0.041	0.780
Acetate, mmol/L	42.598 ^a^	40.438 ^a^	37.065 ^ab^	44.403 ^a^	42.729 ^a^	29.904 ^b^	1.640	0.008	0.004	0.023
Propionate, mmol/L	6.936 ^bc^	9.677 ^a^	9.705 ^a^	7.043 ^abc^	8.080 ^ab^	5.125 ^c^	0.461	0.008	0.004	0.200
Butyrate, mmol/L	6.859	6.909	5.327	6.815	7.577	4.903	0.344	0.182	0.181	0.155
A/P ^2^	5.872 ^a^	4.929 ^ab^	3.908 ^b^	6.525 ^a^	5.397 ^ab^	5.893 ^a^	0.266	0.035	0.260	0.925
Total VFAs ^3^, mmol/L	60.879 ^a^	58.844 ^a^	55.377 ^a^	62.119 ^a^	62.592 ^a^	42.704 ^b^	1.938	0.009	0.003	0.018

^1^ MCP = microbial crude protein; ^2^ A/P = acetate-to-propionate ratio; ^3^ VFAs = volatile fatty acids. ^a–c^ Means in a row not sharing a common letter are significantly different (*p* < 0.05).

**Table 5:** Effects of dietary puerarin supplementation on serum biochemical and antioxidant indices in Hu sheep.

Items ^1^	Puerarin Levels, mg/kg						SEM	*p*-Value		
	0	50	100	200	400	800		ANOVA	Linear	Quadratic
MDA, nmol/mL	5.285 ^bc^	3.129 ^cd^	2.641 ^d^	3.284 ^cd^	6.324 ^b^	7.381 ^a^	0.421	<0.001	<0.001	0.536
GSH-Px, U/mL	254.005	272.131	277.595	297.907	255.858	224.261	12.530	0.640	0.210	0.412
SOD, U/mL	81.490	83.842	80.858	82.929	83.421	81.068	0.498	0.365	0.602	0.220
CAT, U/mL	3.285	5.283	4.092	4.318	5.187	5.327	0.355	0.495	0.195	0.631
BUN, mmol/L	7.249	8.364	7.877	7.863	7.909	8.264	0.161	0.437	0.306	0.908
TP, g/L	69.530	71.679	65.712	67.286	65.222	66.160	0.860	0.207	0.126	0.174
ALB, g/L	25.133 ^c^	25.679 ^bc^	26.903 ^ab^	26.958 ^ab^	27.371 ^a^	26.412 ^abc^	0.238	0.044	0.160	0.005
GLU, mmol/L	4.094 ^c^	4.376 ^bc^	4.780 ^a^	4.523 ^ab^	4.457 ^abc^	4.069 ^c^	0.064	0.004	0.085	0.007

^1^ BUN = blood urea nitrogen; GLU = glucose; ALB = albumin; TP = total protein; MDA = malondialdehyde; CAT = catalase; GSH-Px = glutathione peroxidase; SOD = superoxide dismutase. ^a–d^ Means in a row not sharing a common letter are significantly different (*p* < 0.05).

## Data Availability

The raw sequencing data used in this study were deposited in the NCBI Sequence Read Archive (SRA) under BioProject accession number PRJNA1478048. All data generated or analyzed during this study are included in this published article.

## Abbreviations

The following abbreviations are used in this manuscript:

ADG	Average daily gain
ADMI	Average daily dry matter intake
F/G	Feed-to-gain ratio
DM	Dry matter
CP	Crude protein
NDF	Neutral detergent fiber
ADF	Acid detergent fiber
EE	Ether extract
AIA	Acid-insoluble ash
VFAs	Volatile fatty acids
NH_3_-N	Ammonia nitrogen
MCP	Microbial crude protein
A/P	Acetate-to-propionate ratio
BUN	Blood urea nitrogen
TP	Total protein
ALB	Albumin
GLU	Glucose
CAT	Catalase
SOD	Superoxide dismutase
GSH-Px	Glutathione peroxidase
MDA	Malondialdehyde
TMR	Total mixed ration
PCoA	Principal coordinate analysis
LEfSe	Linear discriminant analysis effect size
KEGG	Kyoto Encyclopedia of Genes and Genomes
SEM	Standard error of the mean
SD	Standard deviation
NE_mf_	Net energy for maintenance and growth
ANCOVA	Analysis of covariance
BW	Body weight
ANOVA	Analysis of variance
OTUs	Operational taxonomic units
LDA	Linear discriminant analysis

## Appendix A

**Table A1:** Adjusted final body weight of Hu sheep after controlling for initial body weight using ANCOVA.

Puerarin Supplementation, mg/kg DM	Adjusted Final BW, kg	SE	95% CI	Overall *p*-Value
0	25.560 ^b^	0.348	24.824–26.297	0.038
50	26.755 ^ab^	0.340	26.033–27.477	
100	27.313 ^a^	0.340	26.591–28.034	
200	26.455 ^ab^	0.339	25.736–27.173	
400	26.783 ^ab^	0.340	26.063–27.503	
800	25.987 ^ab^	0.391	25.157–26.817	

Values are estimated marginal means obtained by ANCOVA, with dietary treatment as the fixed effect and initial BW as the covariate. The experimental unit was the pen, with four replicate pens per treatment. The covariate was evaluated at an initial BW of 21.198 kg. The *p*-value represents the overall effect of dietary treatment. Pairwise comparisons were adjusted using the Bonferroni method. Means with different superscript letters differ significantly (*p* < 0.05); the adjusted final BW was greater in the 100 mg/kg group than in the control group (*p* = 0.039). The dietary treatment × initial BW interaction was not significant (*p* = 0.649). BW, body weight; SE, standard error; CI, confidence interval; ANCOVA, analysis of covariance; DM, dry matter.
